# Delirium in hospitalized COVID-19 patients is associated with dynamic changes in peripheral immune gene expression

**DOI:** 10.1007/s11357-025-01898-x

**Published:** 2025-09-22

**Authors:** Sara C. LaHue, Naoki Takegami, Rubinee Simmasalam, Abiya Baqai, Elena Munoz, Anya Sikri, Thibault du Buisson de Courson, Nilika S. Singhal, Walter Eckalbar, Charles R. Langelier, Carolyn M. Hendrickson, Carolyn S. Calfee, David J. Erle, Matthew F. Krummel, Prescott G. Woodruff, Tomiko Oskotsky, Marina Sirota, Adam Ferguson, Vanja C. Douglas, John C. Newman, Samuel J. Pleasure, Michael R. Wilson, Neel S. Singhal

**Affiliations:** 1 Department of Neurology, School of Medicine, University of California-San Francisco (UCSF), San Francisco, USA; 2 Weill Institute for Neurosciences, UCSF, San Francisco, USA; 3 Buck Institute for Research On Aging, Novato, USA; 4 Department of Neurological Surgery, UCSF, San Francisco, USA; 5 Brain and Spinal Injury Center, Zuckerberg San Francisco General Hospital and Trauma Center, San Francisco, USA; 6 Department of Medicine, UCSF, San Francisco, USA; 7 UCSF CoLabs, UCSF, San Francisco, USA; 8 Division of Infectious Diseases, UCSF, San Francisco, USA; 9 Chan Zuckerberg Biohub, San Francisco, USA; 10 Division of Pulmonary and Critical Care Medicine, UCSF, San Francisco, USA; 11 Zuckerberg San Francisco General Hospital and Trauma Center, San Francisco, USA; 12 Department of Pathology, UCSF, San Francisco, USA; 13 Bakar Computational Health Sciences Institute, UCSF, San Francisco, USA; 14 Department of Pediatrics, UCSF, San Francisco, USA; 15 San Francisco Veterans Affairs Medical Center, San Francisco, USA; 16 Division of Geriatrics, UCSF, San Francisco, USA

**Keywords:** Delirium, COVID19, Critical care medicine, Neurology

## Abstract

Delirium is a neurologic syndrome characterized by inattention and cognitive impairment frequently encountered in medically ill older adults. As a hallmark of age-related brain vulnerability, delirium offers a clinical model to investigate how peripheral immune responses contribute to acute brain dysfunction. Peripheral inflammation is a key trigger of delirium, but the patient-specific immune responses that drive delirium onset and recovery remain poorly understood. This retrospective cohort study of prospectively collected biospecimens examines RNA sequencing from peripheral blood mononuclear cells of adults hospitalized for COVID-19 to better understand patient-specific factors associated with delirium (*n* = 64). Longitudinal transcriptomic analyses highlight persistent immune dysregulation in delirium, marked by increasing expression trajectories of genes linked to innate immune pathways, including complement activation, cytokine production, and monocyte/macrophage recruitment. Genes involved adaptive immunity showed a declining trajectory over time in patients with delirium. Although corticosteroid treatment suppressed some aspects of immune hyperactivation, aberrant responses contributing to delirium were exacerbated. Delirium resolution was characterized by normalization of key transcripts such as *CCL2* and innate immune markers. Novel associations with delirium included transcripts related to stress granule assembly and the T cell regulators *DUSP2* and *KLF10*. Delirium in COVID-19 is associated with distinct and dynamic peripheral immune trajectories that are modulated by corticosteroids. Further understanding these mechanisms has important implications for preventing delirium in older adults. These findings provide novel mechanistic insights with translational relevance for immunomodulatory strategies targeting maladaptive immune responses to prevent or treat delirium in medically ill populations.

**Supplementary Information:**

The online version contains supplementary material available at 10.1007/s11357-025-01898-x.

## Background

Among patients hospitalized for hypoxia due to severe acute respiratory syndrome coronavirus (SARS-CoV)−2 infection, the development of delirium is associated with worse outcomes, especially in older patients [1–3]. Delirium is a common complication of hospitalization in adults and is characterized by an acute change in cognition and fluctuating attention. Over 30% of adults admitted to the hospital for medical conditions or surgery develop delirium during their hospitalization, and up to 70% of patients admitted to the intensive care unit (ICU) develop delirium [4–8]. Delirium is linked to longer hospitalizations and greater morbidity and mortality. Major risk factors for delirium include advanced age, dementia, the use of sedating medications, and acute infection [9]. Identification of patients most likely to benefit from intensive, multicomponent non-pharmacologic delirium prevention programs may further help reduce complications and cost of care [10, 11]. The identification of delirium relies upon clinical observations and bedside assessments, with no reliable diagnostic imaging- or blood-based tests. The heterogenous nature of delirium complicates development of a single blood-based biomarker; however, recent studies have pointed to plausible common pathophysiological mechanisms including exaggerated immunologic responses and increased blood–brain barrier permeability leading to altered neurotransmission [12–14].

Severe COVID-19 provides a model for studying delirium pathophysiology, given the central roles of systemic inflammatory responses, coagulopathy, and endothelial dysfunction. As part of the University of California San Francisco (UCSF) COMET (COVID-19 Multi-phenotyping for Effective Therapies) and the NIAID IMPACC (Immunophenotyping Assessment in a COVID-19 Cohort) studies, serial biospecimens were prospectively collected, providing a unique opportunity to evaluate transcriptomic responses related to delirium over time. To further our understanding of the underlying biological mechanisms associated with the development of delirium in hospitalized patients with COVID-19, we analyzed transcriptomic data from peripheral blood mononuclear cells (PBMCs) isolated from hospitalized patients with COVID-19, with and without delirium, at admission and/or hospital days 4, 7, 14, 21, or 28. We hypothesized that patients who develop delirium would exhibit distinct peripheral immune gene expression profiles compared to those without delirium, and that treatment with corticosteroids would modulate the association between immune transcriptomic signatures and delirium. These rich clinical and biological data enabled the first longitudinal analysis of peripheral inflammation in relation to the onset, persistence, and resolution of delirium, as well as its modulation by corticosteroids. Our findings provide new insights into immune dysregulation in delirium and highlight potential therapeutic targets to improve neurologic outcomes in aging populations following acute illness.

## Materials and methods

### Cohort selection

Adult patients admitted to UCSF Hospitals or Zuckerberg San Francisco General Hospital (ZSFG) with known or presumptive COVID-19 were screened within 3 days of hospitalization for inclusion in COMET/IMPACC. Patients, or a designated surrogate, provided informed consent to participate in the study in accordance with protocols approved by the Institutional Review Board of UCSF and ZSFG (#20–30497). This study includes patients enrolled between April 8, 2020 and May 1, 2021 in the COMET (COVID-19 Multi-immunophenotyping projects for Effective Therapies; https://www.comet-study.org/) and/or IMPACC studies at UCSF. IMPACC and COMET are prospective studies with aligned protocols that aim to describe the relationship between specific immunologic assessments and the clinical courses of COVID-19 in hospitalized patients. For inpatients, clinical data were abstracted from the electronic medical record into standardized case report forms. Patients with COVID-19 confirmed by PCR testing were included in this analysis only if plasma was collected within 24 h of patient admission or at least 3 serial samples were collected during the hospitalization. All participants are identified with a random identification specific for the COMET study after removing variables that could result in patient reidentification.

Incident delirium was defined as new-onset delirium during hospitalization in patients without evidence of delirium at the time of presentation. Delirium was identified using any of the following criteria: (1) a positive Confusion Assessment Method (CAM)-ICU (for patients in the ICU at UCSF or ZSFG); (2) a Nursing Delirium Screening Scale (NuDESC) score ≥ 2 (for patients on the UCSF medical wards); (3) systematic chart review using validated procedures [15]. Chart review encompassed review of daily clinical documentation by physicians and other providers, capturing clinical observations of delirium or encephalopathy that might not have aligned temporally with structured screening assessments to further increase sensitivity. Both CAM-ICU and NuDESC were prospectively assessed by trained nursing staff during each nursing shift [16]. Patients were considered not delirious at presentation if they had a negative first CAM-ICU or NuDESC < 2, and no documentation of delirium or encephalopathy in the admission notes. Delirium resolution was defined as either (a) CAM-ICU remaining negative or NuDESC remaining < 2 for the rest of the hospitalization after a prior positive result, or (b) documentation of delirium resolution in the daily primary team note. The number of days of delirium was defined as the number of days from the initial onset of delirium onset until persistent resolution, hospital discharge, or discharge.

### Biospecimen collection

Peripheral blood was collected at admission and serially throughout the hospitalization (at days 4, 7, 14, 21, and 28, unless discharge/death occurred prior to day 28) in sterile vacutainers after patient consent and transported on ice for processing. Blood plasma was isolated by centrifuging samples at 1000* g* for 10 min at 4 °C, followed by density gradient centrifugation with SepMate PBMC isolation tubes (STEMCELL Technologies). Samples were then aliquoted and stored at −80 °C until analysis.

### RNA isolation and sequencing

RNA was extracted from PBMCs using the Quick RNA MagBead kit (Zymo Research) on a KingFisher Flex system (Thermofisher Scientific) according to the company’s protocol. RNA integrity was measured with the Fragment Analyzer (Agilent) and for samples included in the present study RNA integrity number was > 7, with nearly 90% having a value > 8. Total RNA-sequencing libraries were depleted from ribosomal and hemoglobin RNAs, and generated using FastSelect (Qiagen) and Universal Plus mRNA-seq with Nu Quant (Tecan) reagents. Pooled libraries were PE100 sequenced on an HiSeq4000 or PE150 sequenced on an Illumina NovaSeq 6000 S4 flow cell at the Chan Zuckerberg Biohub SF.

### Bioinformatic and statistical analysis

Data analysis was performed in R using the statistical packages that are specifically mentioned below as well as the packages tidyverse, dplyr, ggplot2, EnhancedVolcano, and volcano3D [17–22]. The raw reads of the fastq files were tested for quality control using the FastQC software and were then aligned to the human reference genome (hg38 from the University of California, Santa Cruz) to summarize the RNA counts [23]. DESeq2 was used for normalization and differential expression analysis of RNA counts [24–26]. Differentially expressed genes were used as the input for gene ontology (GO) analysis with Enrchr-KG using GO: Biological Pathways [27]. CIBERSORTX (cibersortx.stanford.edu) analysis was applied to the Gene expression matrices to impute immune cell fractions based on a signature matrix derived from single cell RNA sequencing of PBMCs classifying expression patterns into 17 cell types [28]. We applied general linear mixed effects model (glmmSeq) adjusted for sequential organ failure assessment (SOFA) and WHO COVID severity scores to the longitudinal transcriptomic data [29]. The number of subjects included at each time point in the generalized linear model are detailed in Supplementary Table 1. No data imputation was performed. Chi-Square and Fisher’s exact tests were used to assess for statistical significance in the observed frequencies of categorical clinical data using Graphpad Prism 10 (La Jolla, CA) and R, respectively.

## Results

### Cohort information

**Fig. 1:**
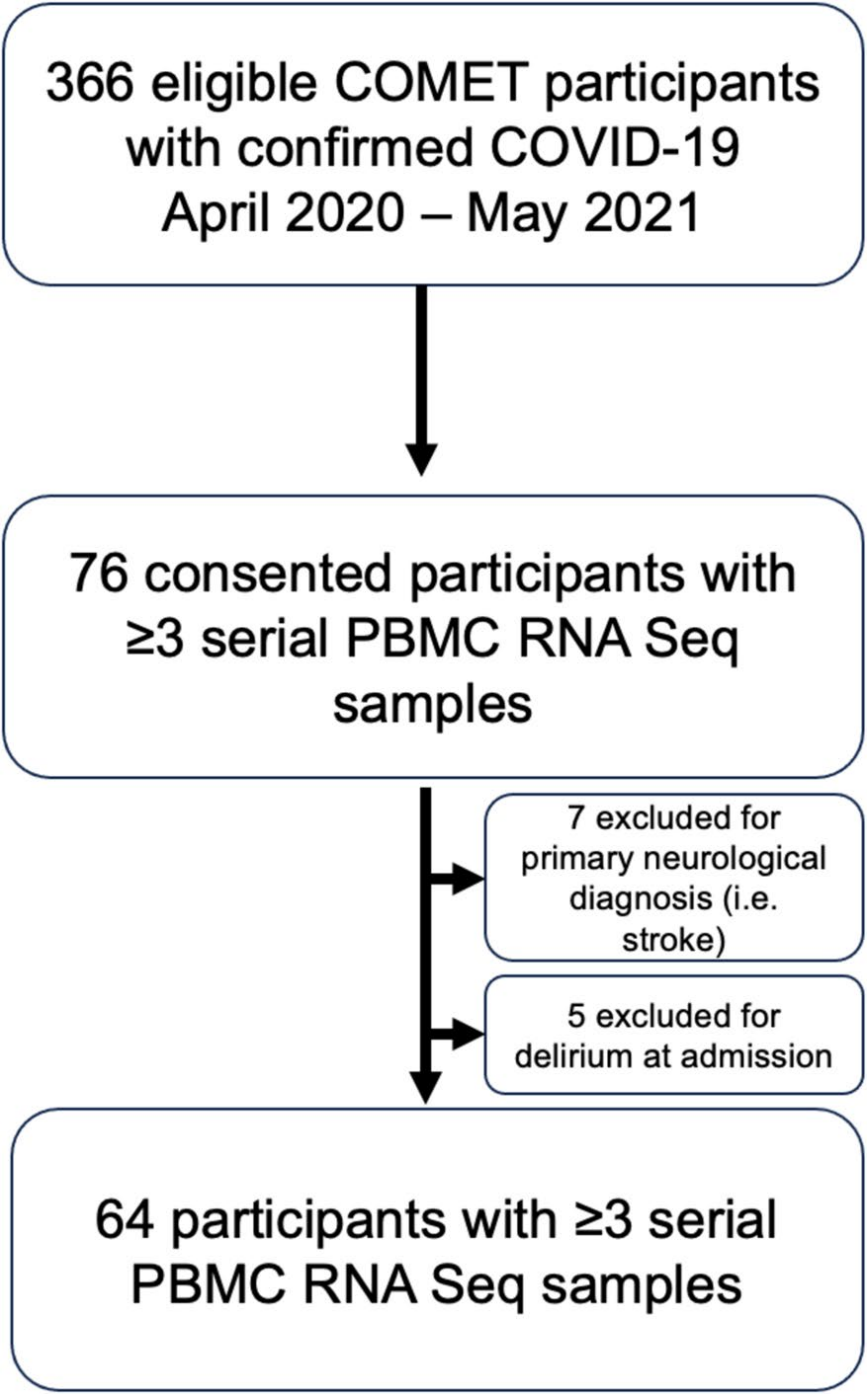
Flowchart of the COMET cohort used for peripheral blood mononuclear cell (PBMC) RNA-sequencing analyses. Patients were classified as having delirium based on positive screening results from nurse administered assessments (Confusion Assessment Method for Intensive Care Unit or the Nursing Delirium Screening Scale) and/or identification of active delirium in physician notes. Patients presenting with delirium or another primary neurological diagnosis were excluded

The COMET cohort contained 64 subjects who did not present with delirium with at least 3 serial samples which were collected at hospital admission and/or hospital days 4, 7, 10, 14, 21, or 28 (Fig. 1A). Delirium was classified based on positive screening on CAM for the ICU, Nu-DESC ≥ 2 for patients not in the ICU, and clinical team notes denoting delirium as an active problem. Clinical characteristics and outcomes among patients with delirium (*n* = 25) and those without delirium throughout the hospitalization (*n* = 39) are shown in Table 1. As expected, patients with delirium presented with more severe disease as indicated by SOFA and WHO COVID severity scores. In addition, length of hospitalization was also generally longer in patients developing delirium. However, a majority of patients in the cohort received ICU care and similar disease-specific treatments. Subsequent analyses comparing patients with delirium and no delirium are presented with adjustment for SOFA and WHO COVID severity classification scores by regression analysis. Additional information regarding delirium subjects, corticosteroid treatment, and onset/resolution of delirium is shown in Supplementary Fig. 1.

**Table 1:** Patient demographics, treatments, and outcomes

	No Delirium (*n* = 39)	Delirium (*n* = 25)	*P*-value
Age, Median (IQR)	58 (47—66)	60 (50- 69)	0.716
Female Sex, N (%)	13 (33.3%)	7 (28.0%)	0.977
Race, N (%)			0.999
American Indian	1 (2.6%)	0 (0.0%)	
Asian or Pacific Islander	6 (15.4%)	4 (16.0%)	
Black	2 (5.3%)	2 (8.0%)	
Other or Multiple Races	22 (56.4%)	14 (56.0%)	
White	7 (17.9%)	5 (20.0%)	
Unknown	1 (2.6%)	0 (0.0%)	
Hispanic or Latino, N (%)	28 (71.8%)	14 (56.0%)	0.642
SOFA score, Median (IQR)	4 (1–10.5)	10 (6–12)	0.023
WHO COVID19 severity score, Median (IQR)	5 (4.5–7)	7 (5–7)	0.030
Received intensive care unit (ICU) care, N (%)	33 (84.6%)	24 (96.0%)	0.570
ICU Length of Stay (days), mean (S.D.)	17.7 (15.9)	24.2 (16.5)	0.125
Total Length of stay (days), mean (S.D)	26.0 (17.1)	33.8 (14.7)	0.064
COVID19 Treatments			
Remdesivir, N (%)	27 (69.2%)	20 (80.0%)	0.824
Convalescent Plasma, N (%)	16 (41.0%)	17 (68.0%)	0.220
Intravenous corticosteroids (IVCS) ≥ 3 days, N (%)	22 (56.4%)	14 (56.0%)	0.923
Dexamethasone (Dex) only	15 (68.1%)	10 (71.4%)	
Methylprednisolone (MP) only	0 (0%)	1 (7.1%)	
Hydrocortisone only	4 (18.2%)	3 (21.4%)	
Dex + Hydrocortisone	1 (4.5%)	0 (0%)	
MP + Hydrocortisone	2 (9.1%)	0 (0%)	
IVCS started prior to hospital day 5	19 (86.4%)	12 (85.7%)	1.000
Reported history of Dementia, N (%)	1 (2.6%)	0 (0.0%)	0.885
In-hospital Death	9 (23.1%)	3 (12.0%)	0.747

### Longitudinal transcriptomic signatures of delirium

**Fig. 2:**
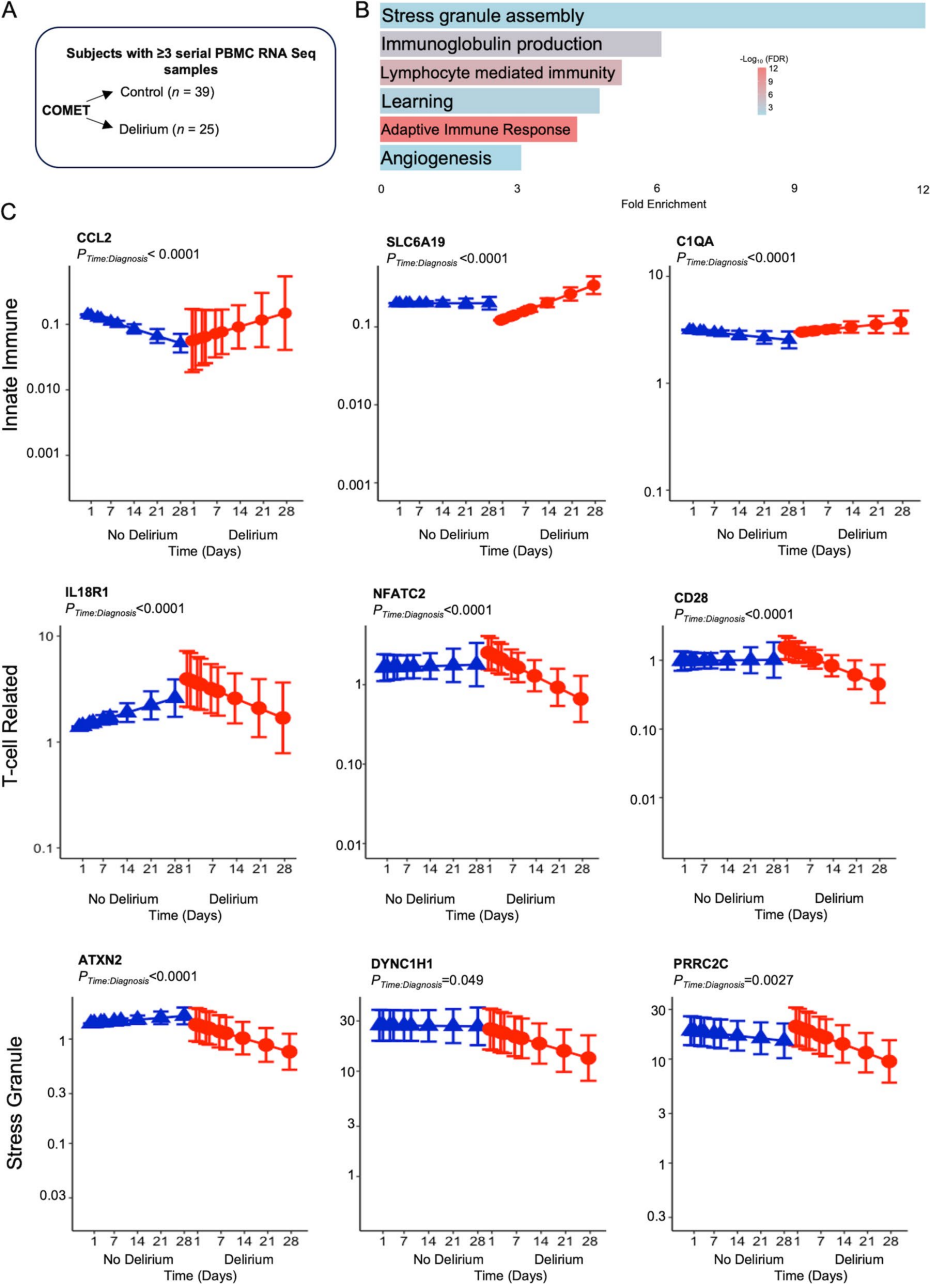
Mixed effects model of serial PBMC sequencing.** (A)** Schematic depicting cohort encompassed by generalized linear mixed-effects modeling (GLMM). **(B)** Gene ontology (GO): Biological pathway (BP) analysis of significantly differentially regulated RNAs in delirium patients over time. GO analysis of genes significant for *time x diagnosis* interaction in GLMM reveals significant regulation of immune-related pathways as well as those regulating stress granule assembly and angiogenesis. **(C)** Plots for normalized expression (*x* axis) of selected Genes with colored points showing regression line of fitted mixed-effects model, with error bars showing 95% CIs (fixed effects)

A unique feature of our study, compared to prior transcriptomic studies in delirium, is the availability of serial sequencing to capture the transcriptomic response in those with delirium compared with those who did not develop delirium. Thus, analyses were performed to compare the transcriptomic trajectory over time in patients with delirium (*n* = 25) versus patients negative for delirium for the duration of hospitalization (*n* = 39). In comparing transcriptomic trajectories over time of delirium patients to patients that did not develop delirium we hope to identify key immune response signatures characteristic of delirium and Generate candidate therapeutic pathways. We included only patients that were hospitalized for at least 1 week and with at least 3 serial samples to provide a robust comparison (Fig. 2A). The number of samples per time point is noted in Supplementary Table 1. We used a general linear mixed effects model (glmmSeq) adjusted for SOFA and WHO COVID severity scores to further dissect these longitudinal molecular signatures at the individual Gene level. Unlike standard RNA sequencing analysis workflows, glmmSeq can fit negative binomial mixed models to allow for Gene expression comparisons over time. Using glmmSeq for the longitudinal analysis, 547 Genes were significantly up- or downregulated in delirium while 357 genes were differentially expressed in delirium over time based on the significance (FDR < 0.05) of the interaction term *time x diagnosis* (Supplementary Data File) and adjusted for disease severity. To further investigate pathway modulation in delirium, genes identified in the adjusted longitudinal mixed-effects model analysis were analyzed for gene ontology (GO): Biological Pathway (BP) enrichment. GO: BP analysis for genes significantly associated with *time x diagnosis* include stress granule assembly, immunoglobulin production, lymphocyte mediated immunity, adaptive immune response, and angiogenesis genes among significantly enriched pathways (Fig. 2B). Interestingly, the GO:BP Learning includes Genes known to be expressed in PBMCs such as neurotensin receptor type 1, sodium-dependent serotonin transporter, neurabin-2, and synaptotagmin-11. Genes for key innate immune and inflammatory mediators demonstrated divergent trajectories over time with initially lower expression levels which increased over time in patients developing delirium compared to controls of C–C motif chemokine ligand (*CCL*)−2, *SLC6A19*, and *C1QA* (Fig. 2C). Similarly, T cell-interacting, activating receptor on myeloid cells (*TARM*)−1, plasma protease C1 inhibitor (*SERPING1*), and complement component receptor-1-like (*CR1L*), which are associated with potentially maladaptive T cell and complement function also demonstrated persistently elevated expression delirium patients over time (Fig. 2C and Supplementary Fig. 2). Interestingly, tumor necrosis factor (*TNF*)-α, was also persistently elevated in delirium patients, but demonstrated a similar trajectory of decreased expression over time (Supplementary Fig. 2). Delirium was also associated with elevated expression of T-cell activating and chemotaxis-related genes in the initial days of hospitalization followed by marked decreases in expression including C-X-C chemokine receptor (*CXCR*)−3, interleukin-18 receptor (*IL18R*)−1, nuclear factor of activated T cells (*NFAT*)-c2, and *CD28* (Fig. 2C and Supplementary Fig. 2). Some genes involved in resolution of immune responses such as prostaglandin D2 receptor (*PTGDR*), *CCR5*, and *CD160* were downregulated in patients with delirium, but demonstrated increases over time in control patients. Interestingly, stress granule-associated genes, ataxin (*ATXN*)−2, cytoplasmic dynein 1 heavy chain (*DYNC1H*)−1, and proline rich coiled-coil (*PRCC*)−2C were significantly down-regulated over time in patients developing delirium which may also contribute to altered immune cell function. In summary, serial analysis of individual patient transcriptomic trajectories supports the hypothesis that an aberrant immune response characterized by initially exuberant innate immune activation and persistently elevated inflammatory mediators distinguish patients with delirium.

**Fig. 3:**
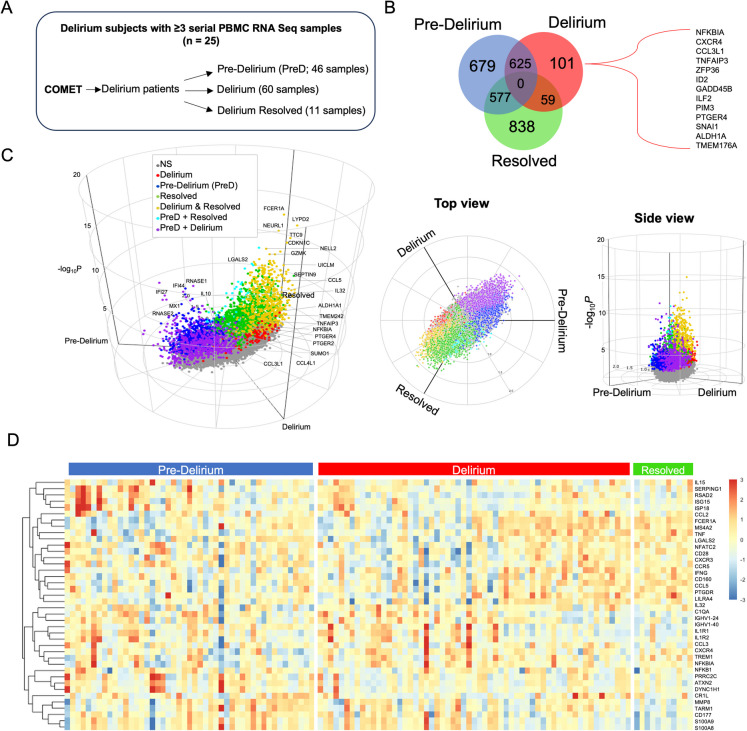
PBMC gene expression of delirium and delirium resolution.** (A)** Schematic of patients and samples included in the analysis. **(B)** Venn diagram demonstrating overlapping genes differentially regulated in samples prior to delirium (pre-delirium), during delirium, and the resolution of delirium. The highlighted gene list includes those also significant in the mixed-effects model analysis in Fig. 2. **(C)** 3D volcano plot demonstrating differentially expressed genes in patients prior to delirium (PreD), during delirium, and after delirium resolution. **(D)** Heat map demonstrating individual patient relative expression levels of top significantly regulated genes at admission and longitudinally in patients with delirium prior to, during, and after the resolution of delirium (*p* < 0.01)

We next examined how the transcriptomic response in patients with delirium varied as a function of time prior to, during, and after delirium resolution (*n* = 25; Fig. 3A). We identified 2879 differentially expressed genes among sequencing before, during, and following delirium (FC > 0, FDR < 0.01; Fig. 3B–D and Supplementary Fig. 3). Out of the Genes differentially expressed in this analysis, 101 were specific to days where patients were positive for delirium, and 102 also overlapped with the *time x diagnosis* interaction in the severity-adjusted analysis above (out of 357). Key immune-related genes that were aberrantly low initially in delirium such as *CCL2*, *PTGDR*, *FCER1A*, and leukocyte immunoglobulin like receptor (*LILR*)-*A4*, increased with the resolution of delirium. Similarly, the resolution of delirium was associated with down-regulation of genes upregulated during delirium such as innate immune (*CR1L*, *SERPING1*, *ISG15*, *S100A8*, and *S100A9*) and inflammatory markers (*NFKBIA, IL18R1*, and immunoglobulin genes; Fig. 3D). These results show aberrant immune responses present at admission in patients that go on to develop delirium also resolve with delirium resolution.

### Earlier onset of delirium is distinguished by innate immune activation

**Fig. 4:**
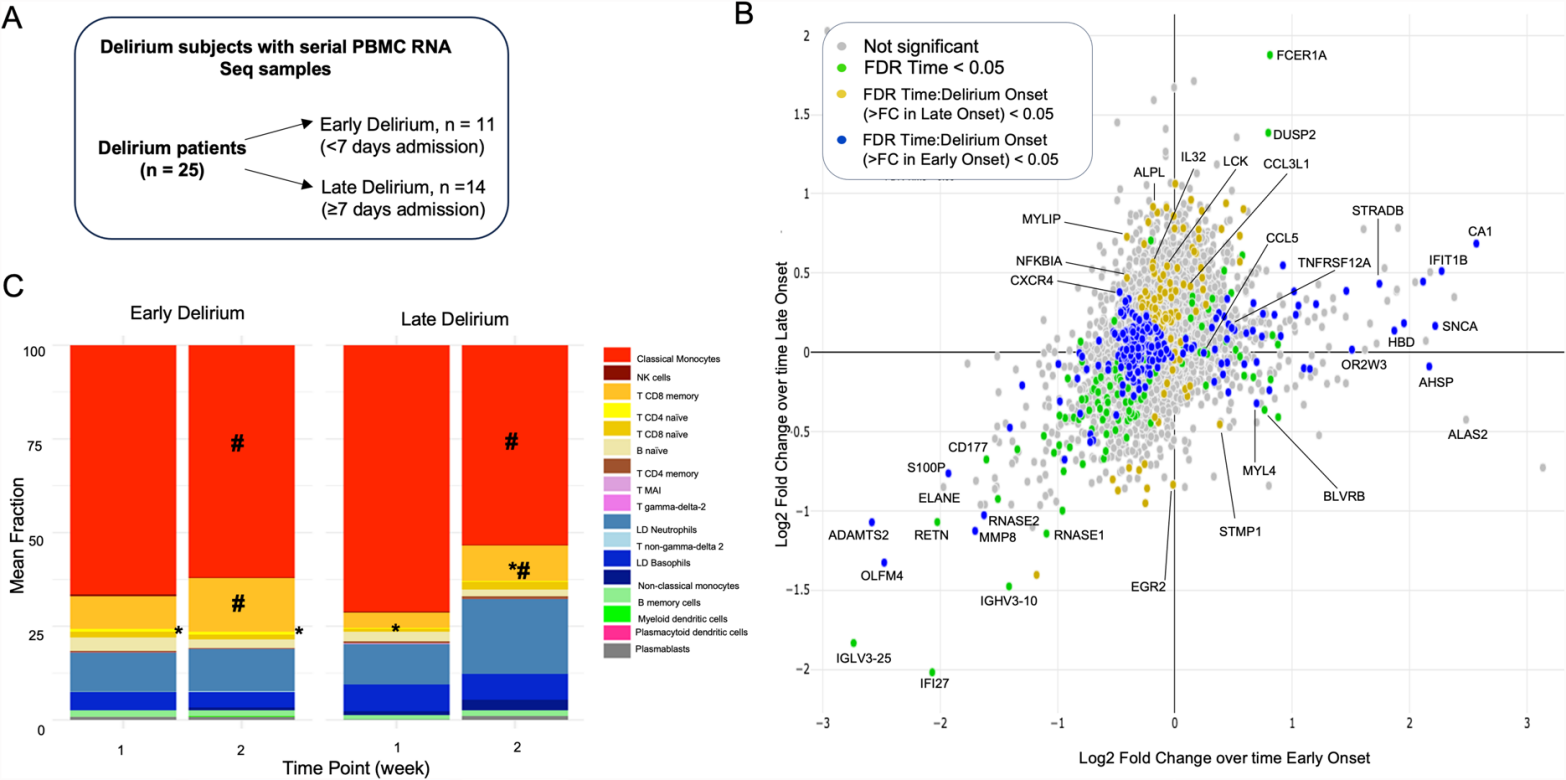
GLM of PBMC gene expression in serial samples in patients with delirium.** (A)** Schematic of delirium patients included in the analysis. **(B)** Scatter plot comparing longitudinal gene expression changes between early (< 7 days, *n* = 11) or late (≥ 7 days, *n* = 14) delirium over one week of paired PBMC RNA seq samples. log_2_ fold change in early and late delirium expression is represented on the *x* and *y* axis, respectively. Genes equally affected by each drug lie along the line of identity (dashed). Fold change and statistical analysis of longitudinal differential gene expression were calculated by negative binomial general linear mixed-effects model. Genes in green show significant (FDR < 0.05) overall change in expression over time; those in blue/yellow show significantly differential change in expression over time between delirium onset based on significant (FDR < 0.05) interaction term *delirium onset* × *time. ***(C)** in silico deconvolution of PBMC fractions using CIBERSORTx demonstrates increased activated CD4 naive T cells in early delirium and decreased CD8 memory T cells in late delirium. Classical monocytes and CD8 memory T cells were also significantly different over time. * indicates main effect for Delirium onset, *p* < 0.05) # indicates main effect for time (*p* < 0.05). LD: low density; MAI: mucosal associated invariant

Mechanisms underlying the development of delirium at later time points (i.e., after days or weeks of illness, treatments, sedation, and sleep disturbances) may be fundamentally different than those contributing to early delirium when acute illness severity is high. When stratified according to late (≥ 7 days, *n* = 14) versus early (< 7 days, *n* = 11) onset of delirium (Fig. 4A), mixed-effects modeling demonstrated contrasting changes in Gene expression over time. Late compared to early onset of delirium had a significant effect on 533 Genes, with 316 showing significant (FDR < 0.05) differential expression change over time. Patients with late onset delirium demonstrated increasing trajectories in *NFKBIA*, lymphocyte-specific protein tyrosine kinase (*LCK*), *IL32*, and *CXCR4*. Late onset delirium was also associated with down-regulation of innate immune markers (*MMP8*, *S100P*), *CD177* (a neutrophil activation marker) compared to patients with early onset delirium (Fig. 4B). In silico deconvolution of PBMC subtype with CIBERSORTx analysis (Fig. 4C) followed by ANOVA revealed significant main effects for delirium onset in naïve CD4 and CD8 memory T cells (*p* = 0.044 and 0.048, respectively) and main effects approaching significance in plasmocytoid dendritic cells. Main effects for timepoint were observed in classical monocytes *(p* = 0.045) and CD8 memory T cells (*p* = 0.024)*.* Post hoc tests further revealed a trend towards significant decrease in classical monocytes over time only in patients with late delirium (Tukey’s HSD, *p* = 0.099).

### Corticosteroid treatment modulates delirium associated gene expression profiles

**Fig. 5:**
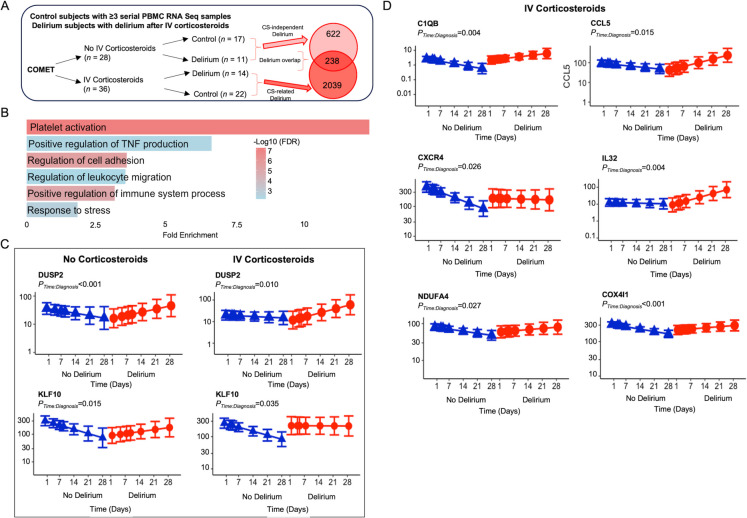
GLM of PBMC gene expression in serial samples separated by corticosteroid administration** (A)** Schematic of patients and samples included in the GLM analysis and Venn diagram demonstrating overlapping genes regulated by the interaction of *time x diagnosis* in patients not treated with iv corticosteroids (top) and those treated with iv corticosteroids (bottom). **(B)** GO:BP analysis of overlapping genes found to be significant for *time x diagnosis* interaction in both patients treated with and without iv corticosteroids. **(C)** Plots for notable genes similarly regulated by delirium regardless of corticosteroid treatment **(D)** Plots for normalized expression (*x*-axis) of selected genes specifically upregulated in patients treated with corticosteroids and developing delirium (red) compared to those not developing delirium (blue). Colored lines show regression line of fitted mixed-effects model, with error bars showing 95% CIs (fixed effects)

Early administration of dexamethasone is a key treatment for COVID19 in patients requiring supplemental oxygen. Methylprednisolone and hydrocortisone were also given at our center for severe COVID-19 infection prior to clinical trials using dexamethasone (see Table 1). Dexamethasone and other corticosteroids modulate immune responses and are a major risk factor for the development of delirium although the underlying mechanisms are not clear [9, 30]. To gain additional insights into mechanisms associated with delirium following IV corticosteroid treatment, we further examined changes in gene expression stratified by corticosteroid treatment and delirium (patients receiving least 3 days of IV corticosteroid treatment and developing delirium following initiation of treatment; Fig. 5A). In patients receiving corticosteroids, severity-adjusted analysis revealed 2039 genes were differentially regulated over time between delirium and control patients (FDR < 0.05, Supplementary Data File). Patients not receiving corticosteroid treatment demonstrated 622 genes significantly altered over time between delirium and control (Supplementary Data File). There was overlap in 238 significantly regulated genes in patients with delirium regardless of corticosteroid treatment. In this overlapping gene set, GO:BP analysis implicated immune and inflammatory gene pathways including platelet activation, TNF production, and leukocyte migration (Fig. 5B). Two genes demonstrated elevated expression over time in patients with delirium compared to non-delirium patients regardless of receiving corticosteroids or not: dual phosphatase specificity (*DUSP*)−2 and Kruppel-like factor (*KLF*)−10 (Fig. 5C). DUSP2 and KLF10 have previously been implicated in immune functions, aging, and metabolic dysfunction. Interestingly, the trajectory of expression of platelet activation (i.e. *ITGB3*, *PF4*) and immune-related (*NFKB1*, *TGFB1*) genes exhibited divergent trajectories in delirium patients dependent on corticosteroid administration (Supplementary Fig. 4). Genes upregulated in delirium patients receiving steroids, but not control patients receiving steroids may be particularly relevant to the pathophysiology of corticosteroid-related delirium in COVID-19, and included complement C1 q B chain (*C1QB*), *CCL5*, *CXCR4*, *IL32*, and numerous nuclear-encoded oxidative phosphorylation genes such as NADH:ubiquinone oxioreductase (*NDUFA*)-*4* and cytochrome c oxidase (*COX*)-*4I1* (Fig. 5D).

## Conclusions

Delirium represents a common and highly morbid manifestation of brain vulnerability in older adults, yet its molecular underpinnings remain poorly understood. Our study directly addresses this gap by characterizing dynamic transcriptomic trajectories in patients with COVID19 who develop delirium, with important implications for aging-relevant immune mechanisms. Our approach differs substantially from previous efforts to characterize peripheral transcriptomic responses associated with delirium in hospitalized patients. First, we analyzed RNA from serial samples of plasma in a well-characterized cohort with a known infection. Second, we performed complementary generalized linear mixed model analyses to further understand how PBMC transcriptomic trajectories differ in patients with delirium, their interaction with delirium onset and corticosteroid treatment, and genes specifically associated with delirium onset and resolution. This high-dimensional readout informs our understanding of the immunopathophysiology of delirium in hospitalized patients with an infectious illness.

While previous work has described associations between delirium and dysregulated immune responses, our findings extend this by highlighting distinct patterns between early and late delirium and the influence of corticosteroid treatment. In severity adjusted analyses, PBMC transcripts classically involved in innate immune responses such as complement pathways, cytokine production, and monocyte/macrophage recruitment exhibited an increased expression trajectory over time in delirium patients (e.g. *CCL2*, *CR1L*, *C1QA*, *TARM*, and *SLC6A19)*. Conversely, genes predominantly involved in adaptive immune functions such as T cell activation (e.g., *CCR5*, CXCR3, *IL18R1*, *CD28*, and *NFATC2*) were initially elevated and then exhibited a decreased trajectory of expression over time in delirium patients. Notably, our analysis also implicated novel pathways such as stress granule assembly in delirium, which may reflect a response to excessive cytokine production and represents an intriguing avenue for further study in delirium [31–33]. Collectively, these findings support the hypothesis that early aberrant peripheral immune responses contribute to later delirium risk in COVID-19.

Analyses of the corticosteroid-related modulation of gene expression also provided intriguing insights concerning the pathophysiology of delirium. Corticosteroid administration profoundly affected several mononuclear cell subpopulations (Supplementary Fig. 4), and notably reduced in silico predicted low density neutrophil fractions after treatment, consistent with potentially beneficial acute effects to reduce inflammation in severe COVID-19 [34–36]. Despite this, delirium patients receiving corticosteroids demonstrated persistent immune dysregulation with prolonged elevations in inflammatory mediators such as *C1qB*, *CCL5*, *IL32*, *CXCR4*, *TGFB1*, and *NFKB1* signaling genes. These markers were comparatively decreased in delirium-free patients not receiving corticosteroids. These findings suggest that corticosteroid-specific immunomodulation may paradoxically contribute to delirium risk while improving outcome from COVID19. Moreover, corticosteroid treatment influenced the metabolic phenotype of PBMCs specifically in delirium patients. Numerous nuclear-encoded mitochondrial genes such as *NDUFA4* and *COX4I1* were upregulated over time compared to control patients, suggesting that altered PBMC metabolism may contribute to persistent immune dysfunction [37]. GO:BP enrichment analysis also implicated modulation of platelet activation in delirium regardless of corticosteroid treatment. Amongst PBMCs, the altered expression of platelet related genes such as *PF4* and *ITGB3* may point to B cell and dendritic cell involvement, which also express these genes [38, 39]. This may represent a promising angle for future research, as platelet count and activity has previously been associated with post-operative delirium [40, 41].

The GLMM analyses also demonstrated that *DUSP2* and *KLF10* have a robust association with delirium regardless of corticosteroid administration. These are also promising targets for future study as *DUSP2* is a critical regulator of signal transducer and activator of transcription (*STAT*)−3 and *DUSP2* activation is known to impair CD4 + helper T (Th)−17 cell differentiation and host defense responses [42–44]. *KLF10* is an important target of *TGF-β* and modulates T cell function and Th17 responses [45, 46]. Taken together, our results point pathophysiological roles for corticosteroids promoting persistent aberrant immune responses which may contribute to delirium risk over time. Further understanding how different etiologies such as infection or medications converge on common pathways such as immune-related (Th17 and TGF*-β* responses), platelet activation pathways, stress granule assembly, and/or mitochondrial pathways to cause delirium is of particular importance for future research.

Our longitudinal sequencing reveals dynamic, aberrant patterns in the initial immune response accompanying delirium with an exuberant innate immune paired with a deficient T cell response that normalizes with the resolution of delirium. Clinically, understanding these temporal patterns may critical for developing novel anti-delirium therapeutics and optimizing the timing of interventions, especially in older patients at risk for delirium. Focusing on PBMCs as in infectious delirium is promising since inflammatory cells and signaling molecules reflect severity of illness and may in turn influence blood–brain barrier permeability and neuronal function [14]. Our results implicate gene pathways known to be involved in delirium such as TNFα and oxidative phosphorylation as well as novel pathways including stress granule assembly, platelet activation, and mediators of TGF-β signaling (DUSP2 and KLF10).

While our study focused on hospitalized patients with COVID-19, many of the immune pathways we identified, including upregulation of innate immunity and suppression of adaptive responses, may be relevant to delirium in the context of other infections. For example, a prior study found enhanced interferon signaling and inflammatory cytokine activity in patients with urinary tract infection-associated delirium, suggesting shared immune activation profiles across infectious etiologies [47]. Nevertheless, COVID-19 presents several unique immunologic features, including dysregulated interferon responses, and widespread corticosteroid exposure, all of which may differentially shape delirium risk or trajectory. These distinctions underscore the importance of future studies that compare peripheral immune signatures across infectious delirium cohorts to identify common versus pathogen-specific mechanisms.

Although this is the largest series of delirium patients with longitudinal PBMC transcriptomic data, a major limitation of this study is the modest sample size, making it difficult to disentangle the contribution of other important delirium-related factors such as age and medications received. While our sample size limits statistical power, particularly in subgroup analyses, we observed statistically significant differences that were robust, biologically plausible, and consistent across multiple time points. These findings likely reflect meaningful immune dysregulation associated with delirium, although further validation of the implicated biological pathways in larger cohorts and complementary experimental models is warranted. Moreover, our institutions use a mix of delirium screening tools to capture episodes of delirium throughout the duration of the hospitalization depending on patient location in the hospital. Although this ensures comprehensive delirium diagnosis across different clinical settings, this approach may have introduced some degree of ascertainment heterogeneity despite high estimated sensitivity for both tools (CAM-ICU ~ 93%, NuDESC ~ 86%). Additionally, we did not differentiate delirium motor subtypes due to both lack of availability of Richmond Agitation-Sedation Scale (RASS) in non-ICU subjects and our modest sample size. Future incorporation of additional standardized assessment measures with structured delirium subtype assessment could offer more detailed insights into patients’ conditions and enable more accurate subclassification of delirium and clarify potential associations with distinct mechanisms. Another limitation regarding interpretation of the longitudinal results is that patients with less severe COVID-19 were discharged sooner and had fewer longitudinal samples available for study, introducing some degree of bias. Distinguishing delirium pathophysiology in patients with longer hospitalizations may be more clinically relevant to future predictive studies. Our study relied on transcriptomic profiling of PBMCs rather than centrally derived biospecimens. The interpretation of our findings should be limited to peripheral immune dynamics which don’t reflect central nervous system–specific immune activity or microglial/astrocytic responses.

In conclusion, this study provides insights from analysis of PBMCs regarding the mechanisms driving delirium development in hospitalized patients with COVID-19. This study identifies persistent innate immune activation, impaired adaptive responses, and mitochondrial and platelet-related dysfunction as transcriptomic features of delirium. Several of the immune signatures we identify, such as loss of CD28 expression, heightened CCL2 and complement activation, mitochondrial gene induction, and platelet activation, closely mirror previously described hallmarks of immune aging [48–50]. Targeting deficient Th17 signaling or hyperactive TGFβ signaling driven by DUSP2 and KLF10 in particular may lead to novel anti-delirium therapeutics. Future work integrating PBMC profiling with markers of biological aging could further delineate mechanisms critical to delirium pathophysiology.

## Supplementary Information

Below is the link to the electronic supplementary material.

Supplementary file1 Supplementary Table 1. Number of subjects included in the generalized linear mixed effects analyses for each time point (DOCX 86.4 KB)

Supplementary file2 Fig. S1: Additional delirium cohort information. Fig. S2: Additional mixed effects gene model of serial PBMC sequencing. Fig. S3: Additional mixed effects gene models of serial PBMC sequencing in patients treated with or without steroids. Fig. S4: CIBERSORTx deconvolution for the effects of IV corticosteroid treatment on PBMC cell fractions (PDF 292 KB)

Supplementary file3 Supplementary Data File (XLSX 16.3 MB)

## Data Availability

RNA-sequencing processed data and metadata can be accessed at Dryad data repository.

## Abbreviations

ATXN-2: Ataxin 2
CCR5: C–C Chemokine Receptor Type 5
CAM: Confusional Assessment Method
CD: Cluster of Differentiation
CIBERSORTx: Cell Type Identification By Estimating Relative Subsets of RNA Transcripts (version x)
C1QA: Complement Component 1, q Subcomponent, A Chain
C1QB: Complement Component 1, q Subcomponent, B Chain
CCL: C–C Motif Chemokine Ligand 2
COMET: COVID-19 Multi-phenotyping for Effective Therapies
COX-4I1: Cytochrome C Oxidase Subunit 4 Isoform 1
CR1L: Complement Component Receptor 1-like
CXCR: C-X-C Chemokine Receptor
DUSP2: Dual-Specificity Phosphatase 2
DYNC1H-1: Cytoplasmic Dynein 1 Heavy Chain 1
FDR: False Discovery Rate
GO:BP: Gene Ontology: Biological Process
HSD: Honest significant difference
ICU: Intensive care unit
IL18R-1: Interleukin 18 Receptor 1
IL32: Interleukin 32
IMPACC: Immunophenotyping Assessment in a COVID-19 Cohort
ITGB3: Integrin Beta-3
IV: intravenous
KLF10: Kruppel-Like Factor 10
LCK: Lymphocyte-Specific Protein Tyrosine Kinase
MMP8: Matrix Metallopeptidase 8
NDUFA-4: NADH:Ubiquinone Oxidoreductase Subunit A-4
NFAT-c2: Nuclear Factor of Activated T Cells, Cytoplasmic 2
NFKB1: Nuclear Factor Kappa B Subunit 1
NFKBIA: Nuclear Factor Kappa B Inhibitor Alpha
NuDESC: Nursing Delirium Scale Score
PBMCs: Peripheral Blood Mononuclear Cells
PF4: Platelet Factor 4
PRCC-2C: Proline Rich Coiled-Coil 2C
PTGDR: Prostaglandin D2 Receptor
S100P: S100 Calcium Binding Protein P
SARS-CoV: Severe Acute Respiratory Syndrome Coronavirus
SERPING1: Plasma Protease C1 Inhibitor
SLC6A19: Solute Carrier Family 6 Member 19
TARM-1: T Cell-Activating Receptor on Myeloid Cells-1
TGFB1: Transforming Growth Factor Beta 1
TNF-α: Tumor Necrosis Factor Alpha
UCSF: University of California- San Francisco
ZSFG: Zuckerberg San Francisco General Hospital
