# Supplementary material for: Delirium in hospitalized COVID-19 patients is associated with dynamic changes in peripheral immune gene expression
Source: GeroScience. 2025 Sep 22;48(3):4209–23. doi: 10.1007/s11357-025-01898-x (PMC13356198; doi:10.1007/s11357-025-01898-x)
Supplement: Supplementary file 1 — Supplementary file1 Supplementary Table 1. Number of subjects included in the generalized linear mixed effects analyses for each time point (DOCX 86.4 KB) [file 11357_2025_1898_MOESM1_ESM.docx]

**Supplementary Table 1.** Number of subjects included in the generalized linear mixed effects analyses for each time point

| **Day** | **No Delirium** | **Delirium** |
| --- | --- | --- |
| **0** | 30 | 24 |
| **4** | 35 | 24 |
| **7** | 36 | 22 |
| **14** | 21 | 20 |
| **21** | 11 | 14 |
| **28** | 7 | 13 |
