# Supplementary material for: Delirium in hospitalized COVID-19 patients is associated with dynamic changes in peripheral immune gene expression
Source: GeroScience. 2025 Sep 22;48(3):4209–23. doi: 10.1007/s11357-025-01898-x (PMC13356198; doi:10.1007/s11357-025-01898-x)
Supplement: Supplementary file 2 — Supplementary file2 Fig. S1: Additional delirium cohort information. Fig. S2: Additional mixed effects gene model of serial PBMC sequencing. Fig. S3: Additional mixed effects gene models of serial PBMC sequencing in patients treated with or without steroids. Fig. S4: CIBERSORTx deconvolution for the effects of IV corticosteroid treatment on PBMC cell fractions (PDF 292 KB) [file 11357_2025_1898_MOESM2_ESM.pdf]

Fig S1

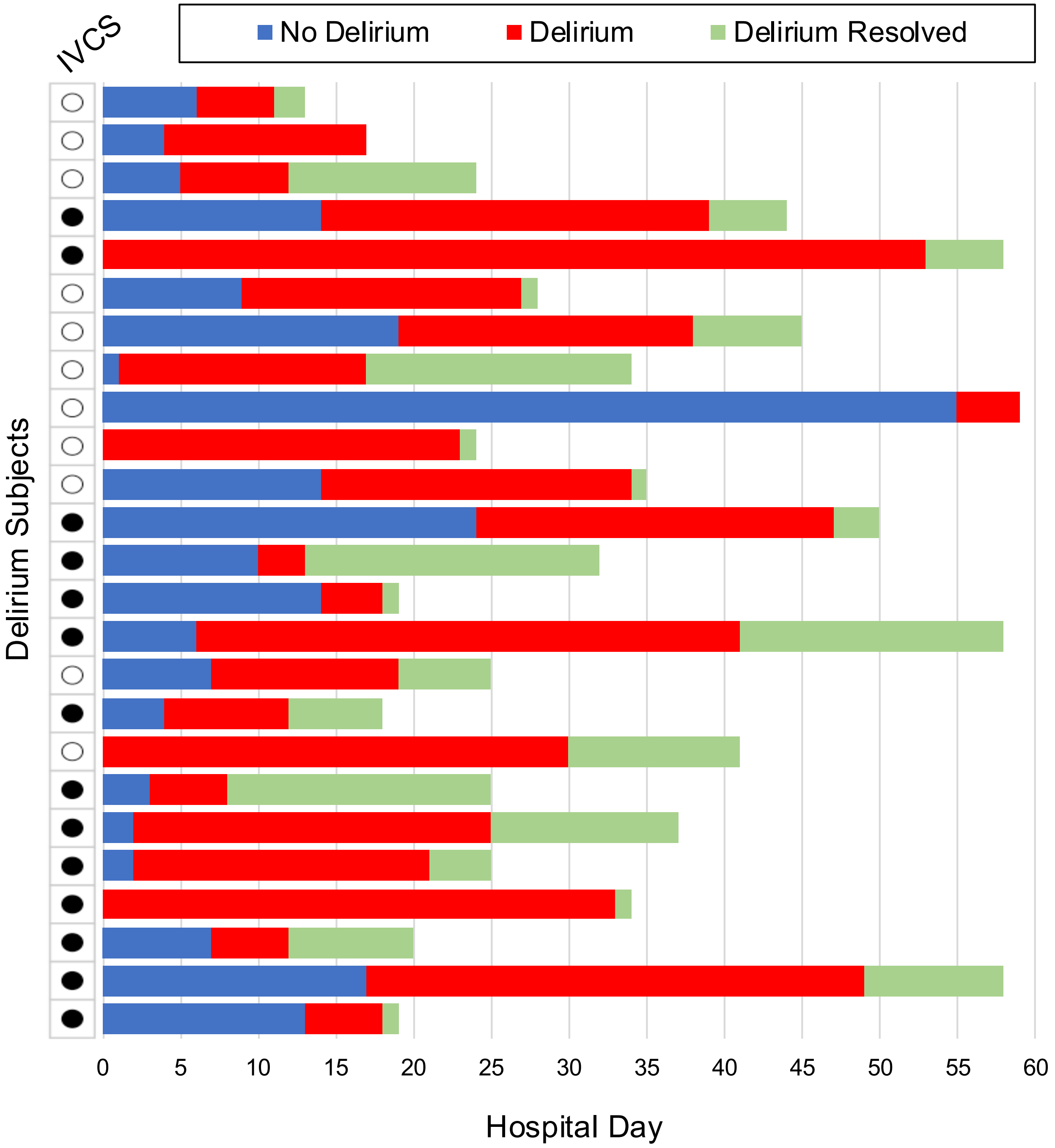

IVCS
No Delirium
Delirium
Delirium Resolved
Delirium Subjects
0
5
10
15
20
25
30
35
40
45
50
55
60
Hospital Day

Fig S2

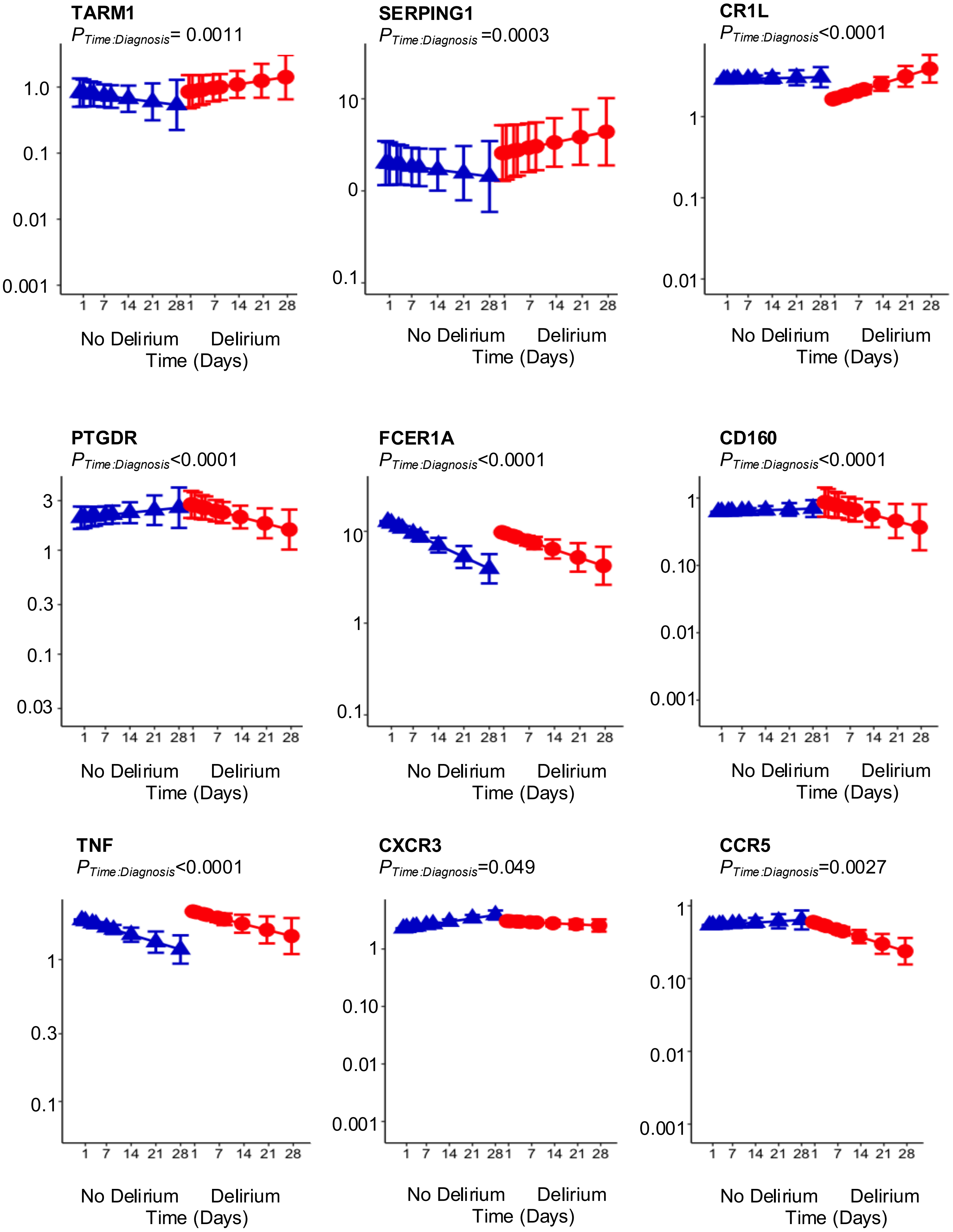

TARM1
P Time:Diagnosis= 0.0011
1.0
0.1
0.01
0.001
1 7 14 21 28 1 7 14 21 28
No Delirium Delirium
Time (Days)
SERPING1
P Time:Diagnosis =0.0003
10
0
0.1
1 7 14 21 28 1 7 14 21 28
No Delirium Delirium
Time (Days)
CR1L
P Time:Diagnosis<0.0001
1
0.1
0.01
1 7 14 21 28 1 7 14 21 28
No Delirium Delirium
Time (Days)
PTGDR
P Time:Diagnosis<0.0001
3
1
0.3
0.1
0.03
1 7 14 21 28 1 7 14 21 28
No Delirium Delirium
Time (Days)
FCER1A
P Time:Diagnosis<0.0001
10
1
0.1
1 7 14 21 28 1 7 14 21 28
No Delirium Delirium
Time (Days)
CD160
P Time:Diagnosis<0.0001
1
0.10
0.01
0.001
1 7 14 21 28 1 7 14 21 28
No Delirium Delirium
Time (Days)
TNF
P Time:Diagnosis<0.0001
1
0.3
0.1
1 7 14 21 28 1 7 14 21 28
No Delirium Delirium
Time (Days)
CXCR3
P Time:Diagnosis=0.049
1
0.10
0.01
0.001
1 7 14 21 28 1 7 14 21 28
No Delirium Delirium
Time (Days)
CCR5
P Time:Diagnosis=0.0027
1
0.10
0.01
0.001
1 7 14 21 28 1 7 14 21 28
No Delirium Delirium
Time (Days)

Fig S3

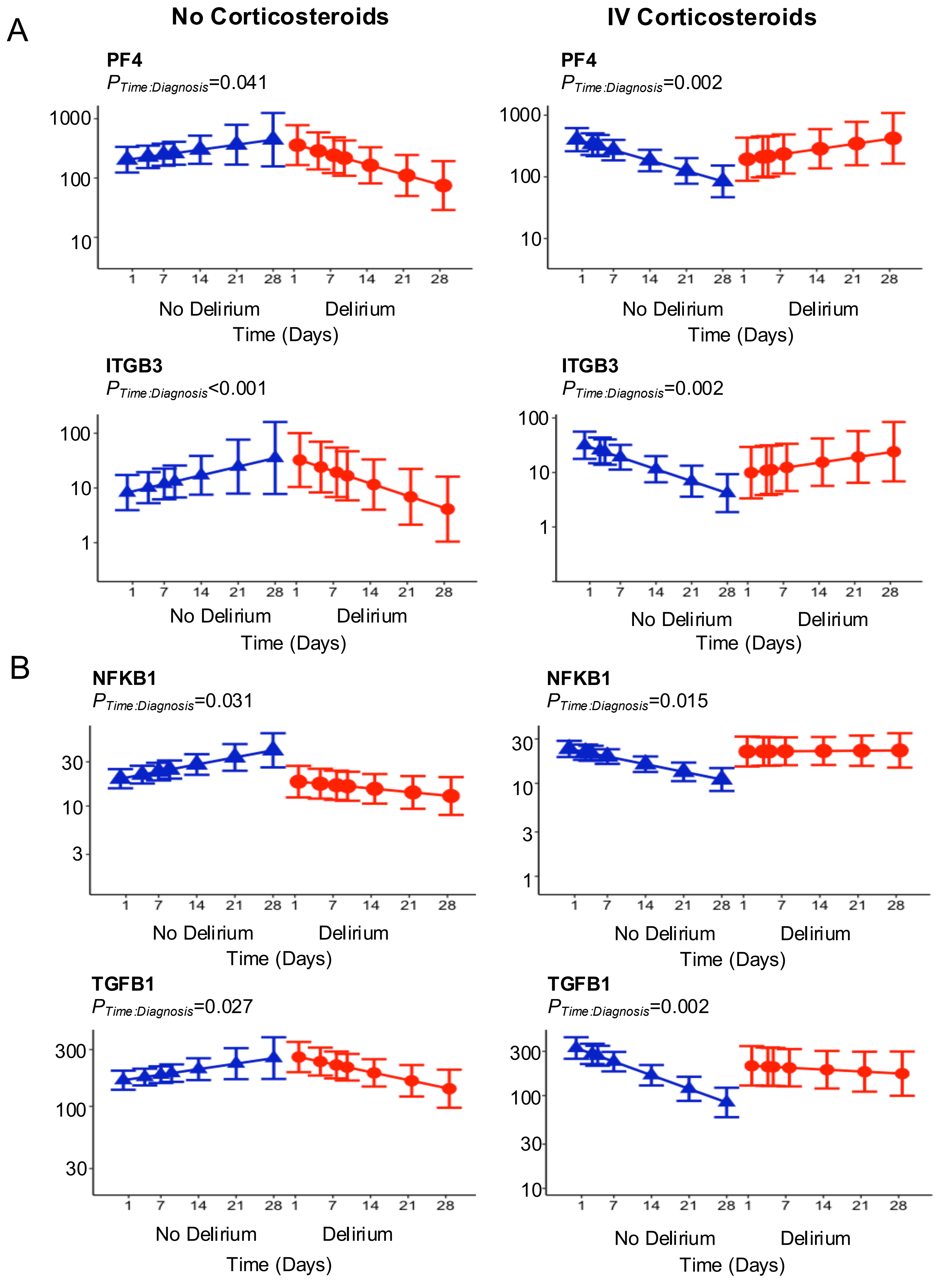

A
No Corticosteroids
IV Corticosteroids
PF4
P Time:Diagnosis=0.041
1000
100
10
1
7
14
21
28
No Delirium
Delirium
Time (Days)
PF4
P Time:Diagnosis=0.002
ITGB3
P Time:Diagnosis<0.001
ITGB3
P Time:Diagnosis=0.002
B
NFKB1
P Time:Diagnosis=0.031
NFKB1
P Time:Diagnosis=0.015
30
3
TGFB1
P Time:Diagnosis=0.027
TGFB1
P Time:Diagnosis=0.002
300

Fig S4

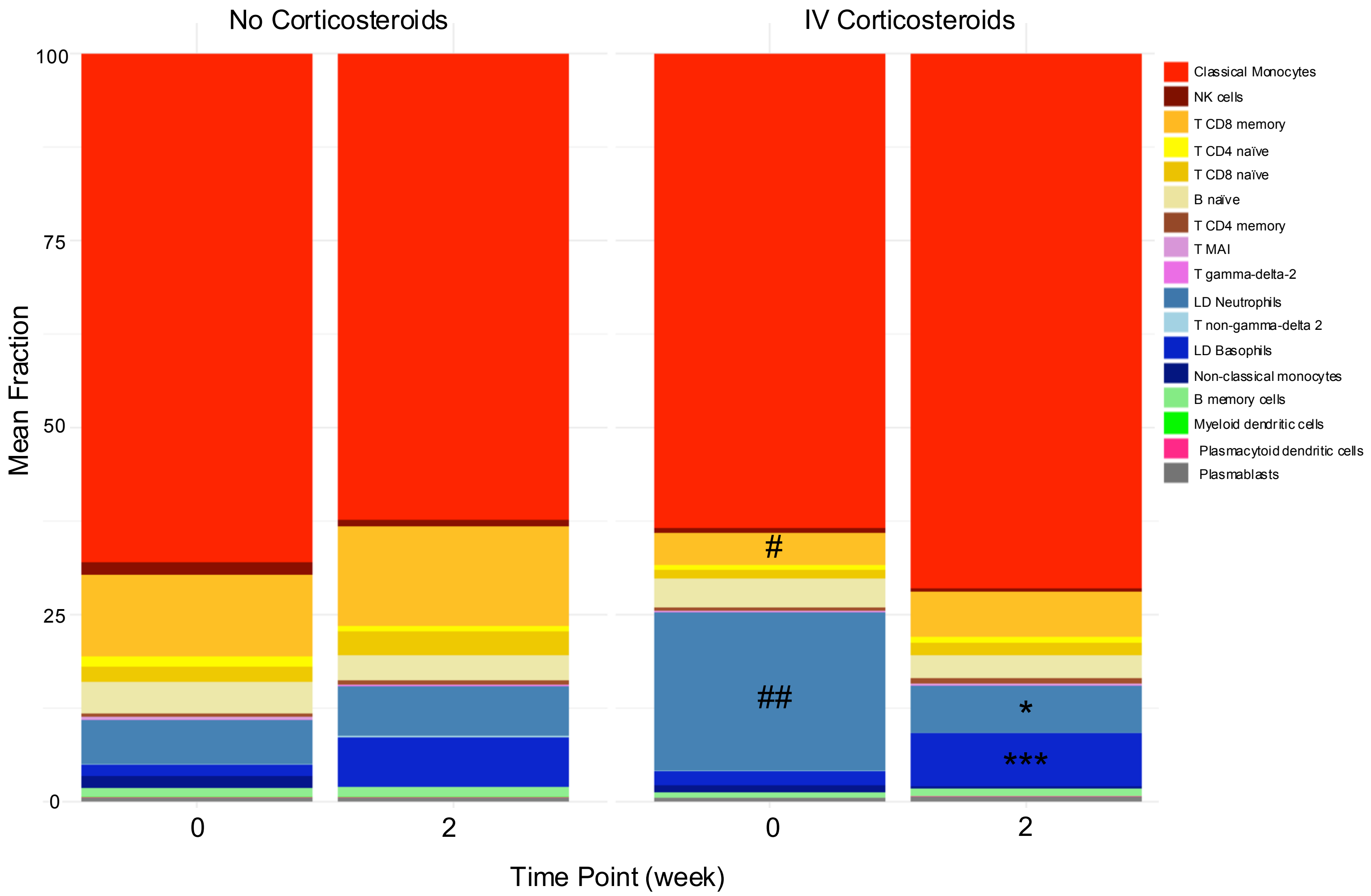

No Corticosteroids
IV Corticosteroids
Mean Fraction
100
75
50
25
0
#
##
*
***
0
2
0
2
Time Point (week)
Classical Monocytes
NK cells
T CD8 memory
T CD4 naïve
T CD8 naïve
B naïve
T CD4 memory
T MAI
T gamma-delta-2
LD Neutrophils
T non-gamma-delta 2
LD Basophils
Non-classical monocytes
B memory cells
Myeloid dendritic cells
Plasmacytoid dendritic cells
Plasmablasts
